# SOCIAL ENGAGEMENT IN OLDER PEOPLE LIVING WITH HIV

**DOI:** 10.1093/geroni/igad104.1996

**Published:** 2023-12-21

**Authors:** Atami De Main, Marshall Glesby, Carrie Johnston, Mark Brennan-Ing, Eugenia Siegler

**Affiliations:** Weill Cornell Medicine, New York City, New York, United States; Weill Cornell Medicine, New York City, New York, United States; Weill Cornell Medicine, New York City, New York, United States; Hunter College, CUNY, New York City, New York, United States; Weill Cornell Medicine, New York City, New York, United States

## Abstract

More than 50% of people with HIV (PWH) are at least 50 years old. PWH experience a high prevalence of multiple chronic conditions and fragile social structures. Although social engagement may be protective against detrimental physical, cognitive, and mental health outcomes, there is limited evidence on older PWH’s social engagement and associated factors in engaging with family, friends, and community. This study sought to examine social engagement in a population of older PWH, using the 13-item Frequency of Leisure Activities Scale from the Research on Older Adults with HIV (ROAH 2.0) study at the Weill Cornell Campus in New York City. We conducted factor and regression analyses to characterize social engagement activities and examine their relationships with AIDS diagnosis, social vulnerability index (SVI), financial strain, loneliness, HIV-related stigma, and depressive symptoms, controlling for demographic factors. PWH (n=349, age range=50-84) were less engaged in social (Mean=1.31; SD=0.71) and cultural-physical (e.g. attending cultural events) (Mean=1.55; SD=0.78) than media-based activities (Mean=3.09; SD=0.72), with Omega reliability estimated at 0.716, 0.651, and 0.620 respectively. Having AIDS, experiencing increased HIV-related stigma, loneliness, and depressive symptoms were associated with low social activity engagement (F(6, 316) = 9.87, p < .001). High SVI score and financial strain were negatively associated with engagement in cultural-physical activities (F(5, 317) = 2.61, p < .001). Increased depressive symptoms were negatively associated with engagement in media-based activities (F(4, 318) = 5.88, p < .001). The findings provide foundations for future research to improve our understanding of social engagement in older PWH.

